# Reexamining the role of *Fusobacterium nucleatum* subspecies in clinical and experimental studies

**DOI:** 10.1080/19490976.2024.2415490

**Published:** 2024-10-12

**Authors:** Madeline Krieger, Mingzhe Guo, Justin Merritt

**Affiliations:** a Division of Biomaterial and Biomedical Sciences, School of Dentistry, Oregon Health & Science University (OHSU), Portland, OR, USA; b Cancer Early Detection Advanced Research Center, Knight Cancer Institute, Oregon Health & Science University (OHSU), Portland, OR, USA; c Department of Molecular Microbiology and Immunology, Oregon Health & Science University (OHSU), Portland, OR, USA

**Keywords:** *Fusobacterium nucleatum*, subspecies, colorectal cancer, microbiome, abscess, periodontitis, inflammophilic, oncobacterium

## Abstract

The Gram-negative anaerobic species *Fusobacterium nucleatum* was originally described as a commensal organism from the human oral microbiome. However, it is now widely recognized as a key inflammophilic pathobiont associated with a wide variety of oral and extraoral diseases. Historically, *F. nucleatum* has been classified into four subspecies that have been generally considered as functionally interchangeable in their pathogenic potential. Recent studies have challenged this notion, as clinical data reveal a highly biased distribution of *F. nucleatum* subspecies within disease sites of both inflammatory oral diseases and various malignancies. This review details the historical basis for the *F. nucleatum* subspecies designations and summarizes our current understanding of the similarities and distinctions between these organisms to provide important context for future clinical and laboratory studies of *F. nucleatum*.

## Introduction

*Fusobacterium nucleatum* (*Fn)* was originally described as being a ubiquitous commensal of the human oral microbiome, where its long, fusiform morphology and promiscuous coaggregation ability are central to its role as a bridge species in the oral biofilm.^1^ However, increasing evidence reveals a more sinister side to *Fn*, as a large number of clinical studies strongly implicate it as a key player in a wide variety of pathological conditions.^1–14^ As with other members of the human microbiome, the mere presence of *Fn* is not necessarily indicative of active disease. In fact, the species is normally found in both healthy and diseased sites of the oral cavity and GI tract. While *Fn* typically exists as a low abundance species in sites of mucosal health, it is evident from numerous studies that elevated levels of *Fn* frequently associate with a variety of adverse health conditions, especially those associated with active inflammation.^1,15^ Although it is still debated whether *Fn* is the causative agent of these conditions (i.e., driver) or if its prominence at sites of inflammation is simply due to a preference for such environments (i.e., passenger), recent molecular work shows that *Fn* has a mechanistic role in disease progression, and it is clear that *Fn* exhibits the typical features of an inflammophilic pathobiont.^16–18^

For both clinical and laboratory investigations, *Fn* is frequently treated as a singular species, even though multiple distinct subspecies of *Fn* have been recognized for decades: *Fn* subsp. *nucleatum*, *Fn* subsp. *animalis*, *Fn* subsp. *polymorphum, Fn*. subsp. *fusiforme*, and *Fn* subsp. *vincentii* (henceforth referred to as *Fnn*, *Fna*, *Fnp*, *Fnf*, and *Fnv*, respectively).^19–22^ In the early literature, *Fnv* and *Fnf* were described as separate subspecies, but they are now known to be genetically identical.^21,23^ *Fnn* has served as the principal laboratory workhorse for the vast majority of *Fn* genetic research, largely due to both historical factors and a common assumption that *Fn* subspecies are relatively synonymous. However, recent evidence examining the subspecies-level distribution of *Fn* at sites of health and disease have challenged this notion. Furthermore, several comparative genomic studies have provided compelling evidence that *Fn* subspecies are actually distinct fusobacterial species.^13,24–28^ However, we will maintain use of the term “subspecies” purely as a historical convention. This review will focus specifically upon our current knowledge of the differences between *Fn* subspecies to help inform future investigations of these ubiquitous human pathobionts.

### The history of Fusobacterium nucleatum subspeciation – how did we get here?

#### Subspecies in the literature

**Figure 1.**
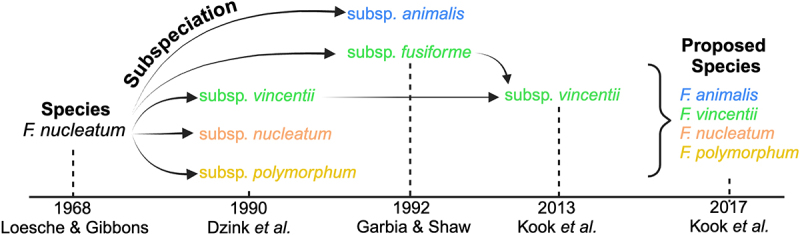
Evolution of species and subspecies level nomenclature assignments for *Fn*. First author and year of publication are shown on the timeline, while the emergence and changes in proposed species and subspecies names are listed above.

**Figure 2.**
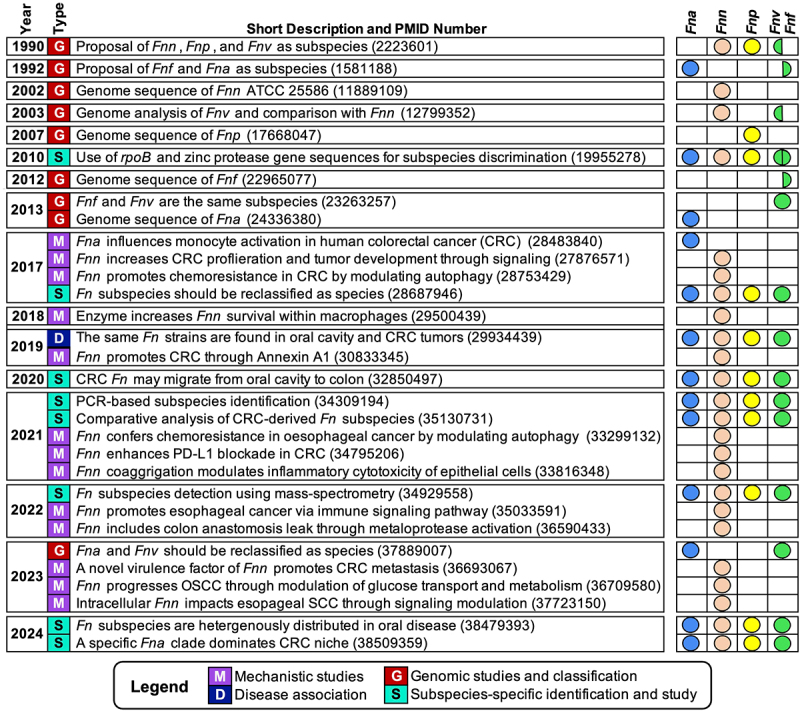
Major milestones in *Fusobacterium nucleatum* subspecies research. Study type is indicated by the color and letter marker in the second column as described in the figure legend. The specific subspecies mentioned in each study is indicated by the colored circles on the right panel (*Fn. animalis* in blue, *Fn. nucleatum* in orange, *Fn. polymorphum* in yellow, and *Fn. fusiforme/vincentii* in green).

Although members of the genus *Fusobacterium* have been the subject of scientific investigation since the 1930s,^29^ the first reference to *Fn* specifically in published literature was from a 1922 German publication.^30^ It is worth noting that the official origin of the “*nucleatum”* species name as well as the *Fn* subspecies names are vague or undocumented in the literature. The next appearance of *Fn* in the scientific record did not occur until 1968 in a study investigating *Fn*’s capacity for amino acid fermentation (Figure 1).^31^ *Fn* was studied sporadically for the next several decades,^32–41^ but it was not until 1990 that a subspecies classification scheme was proposed for *Fn*, when DNA homology and whole-cell protein electrophoretic migration patterns were used to subdivide this species into three newly named subspecies: *Fnp*, *Fnn*, and *Fnv* (Figures 1 and 2).^23^ Despite subsequent unsuccessful attempts to recreate this three-subspecies classification designation using the amino acid utilization profiles of 120 *Fn* strains,^42^ this nomenclature persisted as the accepted classification scheme for *Fn* subspecies.

The first attempt to categorize *Fn* subspecies as either health- or disease-associated occurred in 1990. A study utilizing 32 human oral isolates sorted them into one of the three established subspecies categories based on physiological tests, enzymatic electrophoretic profiles, and DNA hybridization/base composition tests.^43^ This investigation found that a majority of *Fnn* isolates were disease associated, whereas *Fnp* and *Fnv* were more abundant at sites of oral health. Of the three subspecies examined, *Fnv* was found to be the least abundant in either site. Additional early work on *Fn* subspecies also found evidence of subspecies dissimilarities, such as different adhesive properties among strains of *Fnv*, *Fnp*, and *Fnn*.^44^ Amino acid uptake of *Fnf*, *Fnn*, and *Fnp* was found to differ significantly between subspecies, with *Fnf* using the most limited range of amino acid substrates, whereas *Fnn* was found to utilize fewer amino acids than *Fnp* but in higher concentrations.^45^

Although previous work by Gharbia and Shah in 1990 referenced the existence of *Fnf*,^43^ it was not officially added to the classification ranks until 1992 by the same group, along with the newly identified and named organism *Fna* (Figures 1 and 2).^19^ The same work illustrated that biochemical, physiological, and molecular tests could discriminate between these two subspecies. However, this paper also noted an inability to distinguish *Fnv* from *Fnn*. A review published soon after the official classification of *Fna* and *Fnv* provided an early summary of the evidence supporting these changes in nomenclature as well as specific laboratory tests for subspecies discrimination.^46^

As scientists began to scrutinize the newly established four *Fn* subspecies, further questions arose about the appropriate mechanisms needed to distinguish among them, leading some to even question the fundamental clinical and/or biological utility of *Fn* subspecies distinctions. A 1996 study characterized 18 *Fn* isolates utilizing the allozyme electrophoretic profiles of 21 enzyme loci, which revealed two distinct groups inhabiting similar oral niches along with 82.5% similarity among the tested loci.^47^ The following year, the same group used this approach along with physiological and metabolic tests to classify an additional 44 isolates into three categories based upon their isolation site (oral, extraoral, or single unique oral isolate) but not their subspecies.^48^ This paper explicitly argued against the subspecies classification scheme, suggesting that minimal differences exist between *Fna*, *Fnn*, and *Fnv* in terms of physiological and metabolic capabilities, while also asserting a lack of evidence for differences in pathogenicity between the subspecies. Around the same time, a metabolic study of *Fn* isolates grown in chemically defined media concluded that the subspecies classification of *Fn* was unreliable due to the variable results of the recent allozyme electrophoretic enzyme panels.^49^ Similarly, a 1997 review described the subspecies classification scheme as being on “shaky ground”.^50^ Consequently, these publications cast doubt upon the validity of the new four subspecies classification scheme, but in retrospect, this also highlights the challenges often encountered with bacterial taxonomy during the era preceding Next-Generation Sequencing technologies.

Despite the ongoing debate about the validity of *Fn* subspecies classification, proponents of this new four subspecies designation soon outpaced out the early critics. Each of the *Fn* subspecies were mentioned in a 1997 study of clinically relevant anaerobes analyzed using arbitrarily primed PCR (AP-PCR) fingerprinting.^51^ Three *Fn* subspecies were also described as part of a core group of subgingival plaque flora.^52^ The genome sequence of *Fnn* strain ATCC 25586 was first published in 2002 (Figure 2)^53^ and was later employed in a subsequent comparative genomic analysis with the newly identified genome of *Fnv* strain ATCC 49256, resulting in the identification of 118 unique *Fnv* open reading frames (ORFs) (Figure 2).^54^ Furthermore, the July 2002 minutes of the International Committee on Systematics of Prokaryotes Subcommittee clearly indicate that *Fn* subspecies were recognized as distinct organisms, despite prior questions about the validity of subspecies-level classification.^55^ The first draft genome sequence of *Fnf* was published in 2012^56^ before it was later recognized to be synonymous with *Fnv* (Figures 1 and 2).^21^ The first draft genome sequence of *Fna* was subsequently published in 2013 using the strain ChDC F324, which was originally isolated from a subgingival plaque sample (Figure 2).^57^

### Subspecies identification methods

#### Early DNA-based Fn subspecies classification approaches

As advancements in DNA sequencing technologies accelerated in the late 1990s and into the early 2000s, an increased number of clinical studies began differentiating between *Fn* subspecies using genotypic comparisons. One such early attempt employed an AP-PCR approach.^51^ While this study lacked the phylogenetic analysis needed to potentially group the isolates into specific subspecies, the authors concluded that AP-PCR may be a suitable approach to do so in future studies. *Sac*II digestion was also tested as a method to determine subspecies, but this restriction digest proved to be unreliable with clinical isolates. In 2003, Kook *et al*. developed a set of DNA–DNA hybridization probes specifically designed to categorize the subspecies of *Fn*.^58^ Four out of the 96 probes they tested had subspecies specificity, providing an unambiguous diagnostic screen for subspecies identifications.

#### 16S rRNA gene sequence-based Fn subspecies classification approaches

Since its discovery as a species-level determinate in the 1980s,^59^ the 16S rRNA gene sequence has been utilized as a gold standard for bacterial taxonomic classification.^60,61^ Accordingly, 16S-based sequencing methodologies have also been applied to distinguish among *Fn* subspecies, albeit with moderate success due to the low number of 16S rRNA gene single nucleotide polymorphisms (SNPs) among *Fn* subspecies.^62^ In 2002, the first sequences of different fusobacterial 16S-23S internal transcribed spacer (ITS) regions were reported.^63^ These data were used to develop the first phylogenetic tree of *Fn* subspecies in comparison to other *Fusobacterium* species (Table 1). Notably, the tree placed *F. simiae*, *F. naviforme*, and *F. periodonticum* within the *Fn* subspecies cluster, suggesting that the variations between these *Fn* subspecies may be more akin to species-level distinctions. Consequently, the study questioned the appropriateness of the subspecies-level classification scheme originally proposed by Gharbia and Shah in 1992,^19^ but it also refrained from proposing any substantive changes, since the authors concurrently concluded that their ITS data supported the existence of *Fn* subspecies. In a follow up study, a sixth *Fn* subspecies named *Fn* subsp. *canifelium*^64^ was proposed based upon metabolic and biochemical tests of isolates derived from human bite wounds from cats and dogs.^65^ Today, *F. canifelium* is recognized as a distinct *Fusobacterium* species.^66,67^

**Table 1.** Published phylogenetic trees of *Fusobacterium nucleatum* subspecies. All trees showed monophyletic isolation of all *Fn* subspecies.

First Author	Year	PMID	Tree Method	Alignment method	Marker
Gmür, R.	2006	16464693	PHYLIP	ClustalX	16S (bases 54 -938)
Strauss, J.	2008	19114111	MEGA4	ClustalW	16S (bases 8-907) and *rpoB*
Kim, H. S.	2010	19955278	PHYLIP	ClustalW	16S, *rpoB*, and *znpA*
McGuire, M.	2014	25370491	Unlisted	Unlisted	498 orthologous genes conserved across all strains
Ang, M. Y.	2016	27540086	MEGA	Clustal Omega	16S, core-genome SNPs alignments
Kook, J. K.	2017	28687946	MEGA	ClustalW	16S
Richardson, M.	2020	33054632	FastTree	PyNAST	3’ end 16S gene (starting around bp 785) with downstream ITS region removed
Queen, J.	2021	35130731	FastTree	MUGSY	Core genes
Ma, X.	2023	37042769	RAxML-NG	MAFFT	Core proteins determined with OrthoFinder
Krieger, M	2024	38479393	FastTree within Anvi’o	MUSCLE within Anvi’o	275 functionally divergent core genes present in all 72 genomes
Zepeda-Rivera, M.	2024	38509359	kSNP3	MUSCLE using MEGA X	*rpoB* alone; combination of *rpoB*, 16S, *znpA*, *nusA*, *nusG*

Despite exhibiting significant homology between *Fn* subspecies, the 16S V2-V4 region has been successfully utilized for subspecies identification of isolates from CRC tumors.^12^ Similarly, another recent study examined a larger 792 bp fragment mapping to the second half of the 16S gene to identify *Fn* subspecies in the saliva and gastric sites of irritable bowel disease (IBD) patients, allowing the authors to group sequences into subgroups based upon SNPs.^68^ The 16S gene V4-V5 region has also been successfully employed to quantify the composition of actively growing organisms within endodontic infections, where *Fna* was found to be the dominant *Fn* subspecies within the study cohort.^69^

#### Other genomic markers used for Fn subspecies classification

Although 16S gene sequence analysis has remained as the most popular method of classifying phylogeny, the limited heterogeneity among *Fn* 16S gene sequences yields relatively low confidence in its power to distinguish between subspecies.^62^ This has spurred the development of alternative DNA sequence-based classification approaches. Two genes that have proven to be useful in this regard are *rpoB* (encoding the beta subunit of RNA polymerase) and *znpA* (encoding the fusobacterial zinc protease). Both genes have highly conserved primer binding sites that support workflows analogous to the typical 16S-based approaches, but yield a significantly greater number of SNPs, resulting in much higher confidence subspecies resolution compared to traditional 16S genotyping approaches (Figure 2).^62^ For example, a 2008 study evaluated 26 *Fn* and 7 *F. periodonticum* human intestinal tract isolates using a combination of phenotypic, genotypic, and biochemical techniques to determine whether the population of oral fusobacteria differed from the community found in the intestine.^70^ Phylogenic analysis of ~800 bp and 500 bp segments of 16S rRNA and *rpoB* genes, respectively, yielded a tree that grouped isolates into distinct clusters based upon subspecies. Group IV, which clustered *Fna* isolates, contained over half of the collected fecal isolates, implicating *Fna* as the predominant *Fn* subspecies within the gut. More recently, an *rpoB* amplicon-sequencing based approach was utilized to discriminate fusobacterial subspecies within CRC tumor and fecal samples.^13^ This method, termed “Frpo-B seq,” employs a ~ 370 bp segment of the *rpoB* gene to distinguish between various microbial taxa, including *Fn* subspecies. This methodology was able to profile a surprisingly diverse community of microbiota from tumors and healthy tissue, noting that, among *Fn* subspecies, *Fna* and *Fnp* were the most abundant.

The *znpA* gene encoding the fusobacterial zinc protease has similarly proven to be a reliable marker of *Fn* subspecies.^62^ Recent work describing a comprehensive analysis of *Fn* subspecies abundance utilized three separate approaches to analyze *Fn* subspecies from clinical samples.^28^ The first is a subspecies-specific set of five traditional PCR primers that produce differently sized, subspecies-specific DNA amplicons for rapid genotyping of clinical isolates. The second approach is a quantitative assay that employs Next-Generation Sequencing (NGS) of a hypervariable segment of the *znpA* gene to directly classify and quantify *Fn* subspecies within complex clinical specimens. Lastly, the utility of the *znpA* NGS approach was independently assessed and verified using a set of newly designed subspecies-specific qPCR primers.

Aside from the *rpoB* and *znpA* gene sequences, other genomic makers have been used to distinguish *Fn* subspecies. A set of fourteen PCR-primers was developed to aid in the identification of *Fn* strains derived from clinical specimens (Figure 2).^71^ Intriguingly, when this discriminative primer set was applied to fecal and tumor-tissue samples from CRC patients, a heterogeneous stratification of *Fn* subspecies was detected, with a strong enrichment of *Fna* in the CRC group. Although not specifically dedicated to subspecies analyses, it is worth noting that an online repository of *Fusobacterium* genomes called “FusoPortal” provides an interactive database of annotated fusobacterial genomes, including multiple genomes from *Fna*, *Fnv*, *Fnn*, and *Fnp*.^72^ A comprehensive transcriptome map of *Fna*, *Fnp*, *Fnv*, and *Fnn* has been curated and is freely accessible, providing the first complete map of both protein-coding and non-coding RNA abundance during early exponential, mid-exponential, and early stationary growth.^73^ The FusoPortal could be a useful resource to identify additional subspecies-specific genomic and transcriptional markers.

#### Non-genomic markers for subspecies classification

Despite the popularity of DNA-sequencing technologies for *Fn* subspecies classification, other strategies have proven to be similarly successful as well. In 2015, MALDI-TOF mass spectrometry (MS) data were first shown to effectively distinguish among *Fn* subspecies.^74^ Later in 2022, the MALDI-TOF MS approach was further optimized to classify *Fn* subspecies directly from patient saliva specimens (Figure 2).^75^ In addition to MS, antibodies have been employed to characterize the antigen profiles associated with fusobacteria found within Chinese and European clinical specimens of necrotizing ulcerative gingivitis, gingivitis, and periodontitis.^76^ The Chinese and European cohorts differed in the types of *Fn* they contained, with *Fna* and *F. periodonticum* being predominant in Chinese samples, and *Fnn* and *Fnv* prevailing in European samples. Although the reason for this discrepancy was undetermined, it was suggested that the heterogeneous distribution of *Fn* subspecies across different populations could be due to disease states or dietary factors.

### Reassessing the current Fn subspecies classification scheme

The first detailed comparative genomic analysis of *Fn* subspecies was completed in 2016, when an *Fn* pangenome was constructed using the genome sequence data housed at NCBI together with the Prokaryotic Genome Annotation Pipeline.^25^ This study revealed a pangenome consisting of 6,666 total gene clusters, 13% (880) of which constituted core genes and 87% comprised the accessory genome (43% dispensable gene clusters and 44% strain-specific gene clusters). The *Fn* pangenome was subsequently designated as an “open” pangenome by mathematical modeling. This designation, coupled with its large accessory genome, provided compelling evidence that *Fn* subspecies possess much higher genomic diversity than might be expected from closely related subspecies, especially among organisms often treated as relatively synonymous in many clinical and laboratory investigations. Another study investigating the ability of CRC-derived *Fn* subspecies to colonize murine models also profiled the genomic differences between previously sequenced *Fn* genomes (Figure 2).^27^ Consistent with the previous report, *Fn* subspecies genomes clustered tightly into distinct phylogenomic groups, with strain-level variations being relatively minor in comparison to the large distinctions observed between subspecies. This work described a slightly larger core genome than previously reported (1,291 protein coding genes), but it also found a similarly large subset of genes unique to each subspecies. A subsequent study constructed a pangenome using the Roary Pan Genome Pipeline to compare 30 *Fn* genomes comprised of members from each *Fn* subspecies and a previously unidentified strain.^26^ The results revealed a much smaller *Fn* core genome consisting of 517 core and soft-core genes present in at least 28 of the 30 isolates, 3,954 “shell” genes found in between 4 and 28 of the isolates, and 8,277 “cloud” genes present in 4 or fewer of the isolates. Despite the different core genome results determined from these studies, they were all consistent in their findings of substantial genomic diversity between *Fn* subspecies. Additionally, this same analysis found large differences in genome sizes between isolates of the four *Fn* subspecies. *Fnp* and *Fna* isolates had the largest genomes (~2.6 and ~2.5 Mb, respectively), while *Fnv* isolates had the smallest (~2.2 Mb).^26^ This pattern of variable genome size remained consistent for multiple isolates, further supporting the observed genomic distinctions between *Fn* subspecies.

Kook *et al*. were the first group to explicitly argue for a species-level reclassification of *Fn* subspecies (Figures 1 and 2).^24^ Their 2017 study performed a whole-genome average nucleotide identity (ANI) analysis that determined an ANI of <93.01% between subspecies, whereas the ANI within subspecies groups was found to be >96%. Compared to the 96.69% ANI of two unequivocally designated subspecies of *Streptococcus constellatus* (subsp. *pharyngis* SKI1060 and subsp. *constellatus* SK53), the weaker ANI of *Fn* subspecies suggested that their genomic differences warranted reclassification as separate species. The results of this study were also consistent with a prior fusobacterial ANI analysis that reported an ANI 89–93% between *Fn* subspecies.^77^ It is worth noting that ANI values <95-96% are commonly accepted as the threshold required for taxonomic speciation.^78^ A recent comprehensive analysis of multiple *Fusobacterium* species similarly advocated for the reclassification of *Fn* subspecies into separate species.^13^ Their analysis of 157 fusobacterial genomes included both ANI analysis and phylogenetic tree constructions using whole genomes, 16S rRNA genes, and *rpoB* gene sequences. The resulting phylogenetic trees placed the four subspecies into separate branches parallel to other recognized *Fusobacterium* species. Likewise, a recent study by Krieger *et al*. provided multiple independent lines of evidence to support *Fn* subspecies reclassification into separate species.^28^ Their conclusions were derived from a combination of both comprehensive genomic analyses of the four *Fn* subspecies as well as phenotypic evidence derived from paired clinical specimens of patient-matched dental plaque and odontogenic abscesses. The data indicated that the four currently accepted *Fn* subspecies exhibit both genotypic and phenotypic distinctions that are far greater than would be expected from bona fide subspecies. This study and others proposed to retain the current *Fn* subspecies nomenclature as the accepted species designations (i.e., *F. animalis, F. nucleatum, F. polymorphum*, and *F. vincentii*). Results of this and other phylogenetic tree-based studies on *Fn* subspecies are summarized in Table 1.

### Fn subspecies in laboratory investigations

#### Genetic manipulation

Genetic manipulation within the genus *Fusobacterium* has proven to be quite challenging. The first full genome of *F. periodonticum*, a close relative of *Fn* and its subspecies, was published in 2007 in a paper that described the organism as “genetically tractable” due to the large number of horizontally transferred genes that were nearly identical to those found in *Clostridium* and *Salmonella* species (Figure 2).^20^ Despite its genome being theoretically malleable, minimal progress has been reported with *F. periodonticum* genetic manipulation. Recently, a CRISPRi system was successfully employed in *F. periodonticum* and *Fnn*,^79^ potentially paving the way for further gene inactivation studies in *Fn* and related species.

In another promising advance for *Fn* genetic studies, it was recently revealed that *Fnn* exhibits natural competence (exogenous DNA uptake) via its Type IV pili.^80^ This ability potentially provides a convenient strategy to introduce foreign DNA into *Fnn*. It was noted that the efficiency of DNA uptake varied between the two ATCC *Fnn* strains examined. Strain-level variability in transformation efficiency is a common feature of most naturally competent bacteria.^81^ It is not yet clear whether this natural competence ability extends to the other relatively understudied *Fn* subspecies, but such an ability could prove to be a boon for genetic studies of these organisms. In 2005, sonoporation was first reported as a successful strategy to construct a double crossover *fadA* mutant in the *Fnp* strain 12230.^82^ This technique was employed in subsequent studies in 2007^83^ and 2009^84^ to create *recA* deletion and *fadA* complementation strains, respectively. Despite the utility of sonoporation for *Fn* genetic manipulation, electroporation-based transformations remain the most commonly employed technique. This was first documented in 2000 by electroporating the *Escherichia coli-Fn* shuttle vector pFN1 into *Fnp* strain ATCC 10953.^85^ Subsequently, several additional shuttle vectors have been developed for electroporation-based plasmid transformations of *Fnn* strain ATCC 23726.^86^ In this same study, a suicide vector pHS17 was successfully employed to engineer the first targeted fusobacterial chromosomal mutation. Several years later, this same system was used to inactivate two *Fnn* membrane proteins involved in triggering lymphocyte cell death.^87^ A recent strategy using methylated plasmid DNA to bypass the *Fnn* restriction-modification systems was able to greatly increase the efficiency of targeted chromosomal gene knockouts and complementation.^88^ Conceivably, this approach could function similarly in a variety of other fusobacteria as well, provided one were to express the relevant restriction methylase(s). Additionally, it is worth noting that *Fn* transformation has so far only been reported in *Fnn* and *Fnp*.^83,85–87^ The genetic tractability of other *Fn* subspecies has yet to be determined.

#### Coaggregation and biofilm formation

Coaggregation is one of the most famous and well-studied phenotypes of *Fn*. This ability is thought to be a key aspect driving the ecology and structure of oral biofilms due to both the unrivaled breadth of *Fn* coaggregation partners as well as its role in recruiting numerous late colonizing species into the oral biofilm.^89,90^ A 1989 study first examined the characteristics of *Fn* coaggregation with other oral species.^91^ This early work was followed by more detailed examinations of *Fn* coaggregation with specific bacterial species, including *Treponema*, *Helicobacter pylori*, *Porphyromonas gingivalis*^90,92–95^ and many others. Co-occurrence of specific *Fn* subspecies with other bacterial species has been similarly noted in multiple studies.^96,^^97–99^ However, current research suggests that differences in fusobacterial coaggregation profiles are largely determined at the strain level, rather than by species or subspecies levels.^100^

While *Fn* has long been characterized by its ability to coaggregate within a complex polymicrobial environment, each *Fn* subspecies appears to possess a unique biofilm-forming capacity. All *Fn* subspecies except *Fnp* have been demonstrated to form monospecies biofilms *in vitro*,^101^ while *Fnp* is able to form biofilm in the presence of amyloid FadA.^102^ Different *Fn* subspecies were also found to uniquely impact the composition of other species present within a mixed species biofilm model.^103^

### Fn subspecies in systemic disease

#### Fn subspecies in oral disease

Our current understanding of *Fn* pathogenesis is primarily derived from laboratory investigations using *Fnn/Fnp* strains.^16–18,104–111^ Due to extensive coaggregations with many other inflammophilic pathobionts,^90,92–95^ *Fn* has been proposed to play a central role in promoting the formation of dysbiotic oral biofilms.^112^ For example, early work revealed that *Fn* abundance increases significantly during the onset of gingivitis,^52^ which is an inflammatory condition that precedes the chronic inflammation characterizing periodontitis. Thus far, the principal virulence factors shown to promote host cell binding/invasion are FadA and Fap2.^84,113,114^ Interestingly, *Fnn* ATCC 23726 contains four *fadA* orthologs while *Fnp* 12230 appears to encode only one,^114^ indicating that different *Fn* subspecies likely utilize distinct adhesins. *Fnp* 12230 also encodes at least one additional FadA paralog.^82^ Similarly, the ability of FadA to compromise the gingival epithelial barrier may be used as a route for *Fnp* and other subspecies to migrate from the oral cavity to various extraoral sites.^115^ *Fnn*, *Fnv*, and *Fnp* have been shown to significantly increase nuclear factor kappa-B (NF-kB), IL-8, and tumor necrosis factor alpha (TNF-α) transcription in neutrophils, activating an inflammatory response that can trigger tissue destruction.^116^ *Fnn/Fnp* also release endotoxins upon decimation, particularly lipopolysaccharide (LPS), which can activate Toll-like receptors on the surface of gingival epithelial cells and fibroblasts to stimulate periodontal inflammation and bone resorption.^117–119^ In addition, *Fnn* and *Fnp* can secrete a serine protease capable of degrading extracellular matrix proteins, leading to the destruction of periodontal connective tissues and the production of immunoglobulins and complement from the host immune system.^120,121^ Through each of these mechanisms, *Fn* may promote a local inflammatory microenvironment that is conducive for periodontal disease progression.^122^ Although all *Fn* strains can deplete neutrophils at the site of infection via necrotic and apoptotic mechanisms,^116^ it appears that each subspecies varies in their capacities to modulate neutrophil function. For instance, *Fnn* and *Fnp* can completely inhibit superoxide production, while *Fnv* displays only a moderate impact on free radical formation.^116^ Additionally, *Fnp* appears to be more efficient at inducing neutrophil apoptosis compared to *Fnn* and *Fnv*.^116^

*Fn* is frequently detected in inflammatory oral infections, such as gingivitis, pulpitis, peri-implant disease, Vincent’s angina, peritonsillar abscesses, periapical abscesses, and leukoplakia, and is a major contributor to halitosis as well.^123–128^ *Fn* has also been identified as a key pathobiont in the development of periodontitis,^129–131^ and is especially abundant within odontogenic abscesses. While *Fnp* was shown to induce subcutaneous abscesses within an animal model,^132^ until recently, it was unclear whether particular *Fn* subspecies predominate within acute periapical and peritonsillar abscesses.^124,125,133^ This question was recently directly examined using paired dental plaque and odontogenic abscess clinical specimens, revealing a particularly strong bias for *Fna* within the inflammatory abscess environment, whereas it is substantially less prevalent in disease-free dental plaque.^28^ *Fn* has also been detected in higher amounts on the tongues of patients suffering from halitosis, which could be linked with its secretion of various volatile sulfur compounds, such as hydrogen sulfide.^126^ Previous research indicates that H_2_S-producing enzyme profiles differ among *Fn* isolates,^134^ suggesting that the production of sulfur compounds might similarly vary between *Fn* subspecies. While multiple studies have reported an increased abundance of *Fn* and other periodontal pathogens in oral leukoplakia lesions,^127,135^ it is currently unclear whether this association stratifies by *Fn* subspecies. In fact, this is a common theme for many *Fn-*associated oral diseases. Based upon our recent understanding of the important distinctions between *Fn* subspecies and the clear enrichment of *Fna* in oral disease specimens, there is a strong rationale to reexamine the ecological role of *Fn* subspecies in sites of oral health vs. disease.

#### Fn subspecies translocation from the oral cavity to the gastrointestinal tract

Although *Fn* was originally thought to be an obligate oral bacterium, studies have since discovered *Fn* at other sites within the body, especially the gut. While early surveys of *Fn* in fecal samples suggested only a limited and/or transient presence within the gastrointestinal (GI) tract,^136^ advancements in sampling methodologies would soon suggest otherwise. A 2008 study examining both oral and GI *Fn* and *F. periodonticum* isolates were the first to note a striking trend: nearly identical fusobacterial strains were found in both the oral cavities and colons of the same individuals.^70^ This result also added further support for a previous speculation of an oral origin for *Fn* strains present in the amniotic fluid of patients experiencing preterm birth.^137^ A recent study examining fusobacteria from the oral cavity and colon identified 28 unique strains from 381 total sequences (Figure 2).^68^ According to the Chao1 richness index, the oral cavity exhibited a more diverse community of *Fusobacterium* species compared to the gastric or colonic sample sites. A principal component analysis of the fusobacterial populations found in each sample type suggested that the gastrointestinal communities are more closely related than those in saliva. When grouped at the subspecies level, different strains of *Fnv* are significantly enriched in saliva. The levels of *Fna* appear to be maintained from the saliva to the colon, whereas *Fnv* is apparently ineffective at colonizing the colon. This is consistent with previous suggestions that *Fna* is particularly adept at translocating from the oral cavity to other body sites.^138^ The mechanistic basis for this trend has yet to be determined.

#### Adverse pregnancy outcomes (APO) stratify by Fn subspecies

*Fn* is a predominant bacterial species associated with adverse pregnancy outcomes,^138^ and the majority of *Fn* isolates from intrauterine infections are *Fna*, followed by a much smaller proportion of *Fnp*. The aforementioned translocation capability of *Fn* may also explain its presence in amniotic fluid and umbilical cord blood, which is linked with neonatal sepsis and is a potential causative mechanism of pre-term labor.^137,139,140^ In addition, a subgingival plaque isolate of *Fna* was determined to be identical to a strain isolated from the placenta of a preterm stillbirth infant, and this same strain was not found in the supragingival plaque, vagina, or rectum of the patient.^141^ In this particular case, the mother had pregnancy-associated gingivitis in addition to a respiratory infection several days prior to the stillbirth. Thus, it was speculated that the respiratory infection weakened the mother’s immune system, thereby facilitating *Fna* translocation to the placenta. Like *Fna*, matching strains of *Fnp* have also been isolated from maternal oral cavities and preterm neonatal specimens with no detectable vaginal colonization.^142^ For more specific details regarding APO-associated virulence mechanisms of *Fn*, we would refer the reader to the review by Vander Harr *et al*.^143^

Several studies have employed murine models of preterm birth to investigate oral/placental *Fn* translocation. Murine premature birth, stillbirths, and unstained live births were triggered by maternal tail vein injections with *Fnp* strain 12230 at days 16 and 17 of pregnancy (proportional to 28–32 weeks of pregnancy in the human).^2^ Additionally, *Fnp* was shown to establish long-term colonization within the murine placentas despite being efficiently cleared from the liver and spleen within 24 hours. Live *Fnp* cells isolated from the uterus, fetus, and amniotic fluid were determined to have reached these sites via hematogenous transmission. Another study examining the effect of *Fnn*, *Fnp*, *Fnv*, and *Fnf* on parturition in preterm mice similarly found that injections of all *Fn* isolates induced varying degrees of APOs, including pre-term birth and intrauterine growth restriction.^144^

Recent studies have shown that *Fn* infiltration was significantly more frequent in endometrial and endometriotic tissues from patients with endometritis, which suggests *Fn* involvement in APO is likely to be multifaceted^145^. *Fn* has also been linked to endometritis cases.^146^ However, none of these recent studies of endometritis have provided subspecies level resolution. Thus, it is currently unclear whether particular *Fn* subspecies predominate.

#### Fn subspecies implications in colorectal cancer (CRC)

In recent years, there has been a rapidly growing interest in the role of *Fn* within malignant tumors, particularly those of CRC. Multiple studies have reported strong associations of *Fn* with CRC tumors sampled from patient specimens (Figure 2).^18,104,147–154^ Furthermore, a comprehensive examination of >1,000 CRC cases across two U.S. cohort studies concluded that the abundance of *Fn* DNA in CRC tumors correlated positively with increased patient mortality.^152^ However, a common limitation found among many *Fn* – CRC studies is the lack of taxonomic resolution provided to distinguish among *Fn* subspecies. Among the minority of studies that have examined *Fn* subspecies composition in tumors, there appears to be a distinct bias for the presence of *Fna*. A study of the CRC tumor microbiota found a significant enrichment of *Fusobacterium*, and specifically *Fna*, in tumors (Figure 2).^12,155^ Likewise, a profile of *Fn* in CRC tumors using *nusA*, *nusG*, and 16S rRNA gene sequences from almost 2,000 patients found *Fna* to be the only subspecies significantly associated with CRC mortality.^4^ Another recent study profiling the mucosal microbiome of colonic biopsies from CRC patients also found *Fna* to be the predominant subspecies within *Fn*-positive CRC tumors.^156^ A MALDI-TOF based technique has similarly been employed to determine the *Fn* subspecies composition within colorectal cancers.^75^ Of the 196 *Fn* proteins identified, 157 were attributed to specific subspecies, with *Fna* comprising the largest fraction, followed by *Fnv* and *Fnp*. *Fnn* proteins were not detected in the adenoma specimens and were present in only half of the control samples. This finding is consistent with another recent analysis of CRC samples that identified an increased abundance of *Fna* and *Fnp* within tumors, with *Fnn* being the least abundant.^13^ A transcriptomic analysis of 807 CRC tumors reported *Fna* to have the most significant association in mesenchymal CMS4 tumors, which typically have a worse prognosis compared to other subtypes.^8^ *Fna* has also been reported to selectively adhere to CRC cells, presumably promoting its colonization and proliferation.^9^

Several recent prominent studies have highlighted the importance of considering subspecies-specific taxonomic resolution in *Fn* research. Zepeda-Rivera *et al*. found that *Fna* not only comprise the majority of *Fn* subspecies detected within a CRC cohort but a specific clade within *Fna* dominates the CRC niche (Figure 2).^14^ Subsequent work by Qin *et al*. found a higher prevalence of *Fna* than other *Fn* subspecies in a CRC cohort, also noting that *Fna* isolates had a higher rate of genome coverage detection within the CRC samples when compared to other *Fn*.^155^ It is worth noting that this study adopted a species-level distinction when referring to *Fna* as “*F. animalis*”, whereas all other *Fn* isolates were called “*F. nucleatum*.”

This recent body of work implicates *Fna* as the most clinically relevant subspecies associated with CRC. However, *Fnn* or *Fnp* strains have thus far been employed in the majority of *Fn* laboratory studies investigating CRC initiation and progression. *Fnn* has been shown to activate NF-kB signaling and stimulate chemokine CCL20 secretion through miRNA modulation in CRC cells.^157^ Additionally, *Fnn* selectively recruits immunosuppressive myeloid suppressor cells (MDSCs) via activation of the NLRP3 inflammasome, resulting in weakened anti-tumor immunity. Three studies using *Fnp* 12230 have demonstrated that the aforementioned adhesin FadA plays an important role in accelerating the growth of CRC cells. A 2013 study reported that FadA facilitates CRC progression by modulating E-cadherin/β-catenin signaling.^158^ Later in 2019, FadA was shown to work in concert with Annexin A1 to activate β‐catenin in CRC cells, thus accelerating cancer growth.^110^ An excreted amyloid-like form of FadA was also shown to promote CRC progression by serving as a scaffold for biofilm formation and host cell adherence.^159^ Additionally, this study found that production of FadA increases acid tolerance, which may be an important mechanism to facilitate GI translocation. It will be interesting to determine whether these same pro-oncogenic mechanisms and others occur similarly with *Fna* or if this organism triggers an altogether distinct response from *Fnn* and *Fnp*.

#### The role of Fn subspecies in other types of tumors is largely unexplored

In addition to CRC, *Fn* appears to have similar associations with various other tumor types throughout the body.^160^ Recent studies have identified an enrichment of *Fn* in oral cancer, but there is still an overall dearth of evidence describing *Fn* subspecies associations among head and neck cancers (HNCs).^5^ A recent meta-analysis of 17 publications reported *Fnn* as the most prevalent subspecies within HNC,^161^ but given the limited amount of supporting evidence, it will be interesting to observe whether this trend holds as additional studies are performed in the future. Similarly, a potential role for *Fn* in breast and pancreatic cancers has been recently reported.^106,162–164^ Additionally, *Fn* associates with both gastric and esophageal cancers and may even serve as a predictor of disease severity.^150,165,166^ Thus far, all of the laboratory studies of *Fn* with these malignancies have employed *Fnn* strains.^16,106,108,109,162–164^

## Discussion

Since *Fn*-associated diseases overwhelmingly occur in a polymicrobial context, a critical question inevitably arises regarding the role of this organism as a driver or passenger. In support of the former, studies have elucidated specific molecular mechanisms underlying *Fn* pathogenicity^17,107,110,111,167^ as well as its role in chemoresistance^18,108^ and immunomodulation (Figure 2).^109^ The majority of such studies have employed model systems incorporating *Fnn*, with a much smaller proportion employing *Fnp*. It is important to note that despite the large number of oral and extraoral conditions associated with *Fn* infection, there is currently limited clinical evidence provided to clearly delineate *Fn* subspecies prevalence in many of these conditions. Current evidence has revealed that substantial biases can exist among the *Fn* subspecies associated with such conditions, which has important implications for the experimental models created to investigate relevant *Fn* pathobiology mechanisms. Furthermore, there is compelling evidence that *Fn* subspecies are in fact distinct *Fusobacterium* species,^13,24,28,77^ and therefore, should be treated as such in laboratory and clinical investigations.

Accordingly, a recent trend toward species-level distinctions has already begun in experimental and taxonomic work, with Kook *et al*. issuing a 2021 correction to their 2017 study to elevate the ranks of *Fna*, *Fnp*, and *Fnv* from the subspecies to species level.^168^ The List of Bacterial Names with Standing in Nomenclature (LPSN) database has made updates to reflect this shift toward species-level distinctions as well. However, LPSN still currently lists all historical *Fn* subspecies under species *F. nucleatum*,^169^ which can lead to confusion. To better clarify these issues, the current review has summarized the known physiological and genomic distinctions between *Fn* subspecies in hopes of promoting further nuanced investigations of fusobacterial roles in human health and disease.

## References

[1] Han YW. Fusobacterium nucleatum: a commensal-turned pathogen. Curr Opin Microbiol. 2015;23:141–19. doi:10.1016/j.mib.2014.11.013. 25576662 PMC4323942

[2] Han YW, Redline RW, Li M, Yin L, Hill GB, McCormick TS. Fusobacterium nucleatum induces premature and term stillbirths in pregnant mice: implication of oral bacteria in preterm birth. Infect Immun. 2004;72(4):2272–2279. doi:10.1128/IAI.72.4.2272-2279.2004. 15039352 PMC375172

[3] Fan Z, Tang P, Li C, Yang Q, Xu Y, Su C, Li L. Fusobacterium nucleatum and its associated systemic diseases: epidemiologic studies and possible mechanisms. J Oral Microbiol. 2023;15(1):2145729. doi:10.1080/20002297.2022.2145729. 36407281 PMC9673791

[4] Borozan I, Zaidi SH, Harrison TA, Phipps AI, Zheng J, Lee S, Trinh QM, Steinfelder RS, Adams J, Banbury BL, et al. Molecular and pathology features of colorectal tumors and patient outcomes are associated with Fusobacterium nucleatum and its subspecies animalis. Cancer Epidemiol Biomarker Prev. 2022;31(1):210–220. doi:10.1158/1055-9965.EPI-21-0463. PMC8755593 34737207

[5] McIlvanna E, Linden GJ, Craig SG, Lundy FT, James JA. Fusobacterium nucleatum and oral cancer: a critical review. BMC Cancer. 2021;21(1):1212. doi:10.1186/s12885-021-08903-4. 34774023 PMC8590362

[6] Sun C-H, Li B-B, Wang B, Zhao J, Zhang X-Y, Li T-T, Li W-B, Tang D, Qiu M-J, Wang X-C, et al. The role of Fusobacterium nucleatum in colorectal cancer: from carcinogenesis to clinical management. Chronic Dis Transl Med. 2019;5(3):178–187. doi:10.1016/j.cdtm.2019.09.001. 31891129 PMC6926109

[7] Taher HJ, Kamel FH. Prevalence and phylogenetic analysis of Fusobacterium nucleatum and virulent factor FadA among ulcerative colitis precancerous and colorectal carcinoma patients in the Iraqi Kurdish population. Arch Razi Inst. 2023;78(1):445–452. doi:10.22092/ARI.2022.360307.2570. 37312686 PMC10258296

[8] Younginger BS, Mayba O, Reeder J, Nagarkar DR, Modrusan Z, Albert ML, Byrd AL. Enrichment of oral-derived bacteria in inflamed colorectal tumors and distinct associations of Fusobacterium in the mesenchymal subtype. Cell Rep Med. 2023;4(2):100920. doi:10.1016/j.xcrm.2023.100920. 36706753 PMC9975273

[9] Zhang H, Jin K, Xiong K, Jing W, Pang Z, Feng M, Cheng X. Disease-associated gut microbiome and critical metabolomic alterations in patients with colorectal cancer. Cancer Med. 2023;12(14):15720–15735. doi:10.1002/cam4.6194. 37260140 PMC10417192

[10] Komiya Y, Shimomura Y, Higurashi T, Sugi Y, Arimoto J, Umezawa S, Uchiyama S, Matsumoto M, Nakajima A. Patients with colorectal cancer have identical strains of Fusobacterium nucleatum in their colorectal cancer and oral cavity. Gut. 2019;68(7):1335–1337. doi:10.1136/gutjnl-2018-316661. 29934439 PMC6582823

[11] Abed J, Maalouf N, Manson AL, Earl AM, Parhi L, Emgård JEM, Klutstein M, Tayeb S, Almogy G, Atlan KA, et al. Colon cancer-associated Fusobacterium nucleatum may originate from the oral cavity and reach colon tumors via the circulatory system. Front Cell Infect Microbiol. 2020;10:400. doi:10.3389/fcimb.2020.00400. 32850497 PMC7426652

[12] Ye X, Wang R, Bhattacharya R, Boulbes DR, Fan F, Xia L, Adoni H, Ajami NJ, Wong MC, Smith DP, et al. Fusobacterium nucleatum subspecies animalis influences proinflammatory cytokine expression and monocyte activation in human colorectal tumors. Cancer Prev Res (Phila). 2017;10(7):398–409. doi:10.1158/1940-6207.CAPR-16-0178. 28483840

[13] Bi D, Zhu Y, Gao Y, Li H, Zhu X, Wei R, Xie R, Cai C, Wei Q, Qin H. Profiling Fusobacterium infection at high taxonomic resolution reveals lineage-specific correlations in colorectal cancer. Nat Commun. 2022;13(1):3336. doi:10.1038/s41467-022-30957-6. 35680952 PMC9184491

[14] Zepeda-Rivera M, Minot SS, Bouzek H, Wu H, Blanco-Míguez A, Manghi P, Jones DS, LaCourse KD, Wu Y, McMahon EF, et al. A distinct Fusobacterium nucleatum clade dominates the colorectal cancer niche. Nature. 2024;628(8007):424–432. doi:10.1038/s41586-024-07182-w. 38509359 PMC11006615

[15] Kunzmann AT, Proença MA, Jordao HW, Jiraskova K, Schneiderova M, Levy M, Liska V, Buchler T, Vodickova L, Vymetalkova V, et al. Fusobacterium nucleatum tumor DNA levels are associated with survival in colorectal cancer patients. Eur J Clin Microbiol Infect Dis. 2019;38(10):1891–1899. doi:10.1007/s10096-019-03649-1. 31367996 PMC6778531

[16] Li Y, Xing S, Chen F, Li Q, Dou S, Huang Y, An J, Liu W, Zhang G. Intracellular Fusobacterium nucleatum infection attenuates antitumor immunity in esophageal squamous cell carcinoma. Nat Commun. 2023;14(1):5788. doi:10.1038/s41467-023-40987-3. 37723150 PMC10507087

[17] Yang Y, Weng W, Peng J, Hong L, Yang L, Toiyama Y, Gao R, Liu M, Yin M, Pan C, et al. Fusobacterium nucleatum increases proliferation of colorectal cancer cells and tumor development in mice by activating toll-like receptor 4 signaling to nuclear Factor−κB, and up-regulating expression of MicroRNA-21. Gastroenterology. 2017;152(4):851–866.e24. doi:10.1053/j.gastro.2016.11.018. 27876571 PMC5555435

[18] Yu T, Guo F, Yu Y, Sun T, Ma D, Han J, Qian Y, Kryczek I, Sun D, Nagarsheth N, et al. Fusobacterium nucleatum promotes chemoresistance to colorectal cancer by modulating autophagy. Cell. 2017;170(3):548–563.e16. doi:10.1016/j.cell.2017.07.008. 28753429 PMC5767127

[19] Gharbia SE, Shah HN. Fusobacterium nucleatum subsp. fusiforme subsp. nov. and Fusobacterium nucleatum subsp. animalis subsp. nov. As additional subspecies within Fusobacterium nucleatum. Int J Syst Bacteriol. 1992;42(2):296–298. doi:10.1099/00207713-42-2-296. 1581188

[20] Karpathy SE, Qin X, Gioia J, Jiang H, Liu Y, Petrosino JF, Yerrapragada S, Fox GE, Haake SK, Weinstock GM, et al. Genome sequence of Fusobacterium nucleatum subspecies polymorphum — a genetically tractable Fusobacterium. PLOS ONE. 2007;2(8):e659. doi:10.1371/journal.pone.0000659. 17668047 PMC1924603

[21] Kook J-K, Park S-N, Lim YK, Choi M-H, Cho E, Kong S-W, Shin Y, Paek J, Chang Y-H. Fusobacterium nucleatum subsp. fusiforme Gharbia and Shah 1992 is a later synonym of Fusobacterium nucleatum subsp. vincentii Dzink et al. 1990. Curr Microbiol. 2013;66(4):414–417. doi:10.1007/s00284-012-0289-y. 23263257

[22] Lawson PA, Gharbia SE, Shah HN, Clark DR, Collins MD. Intrageneric relationships of members of the genus Fusobacterium as determined by reverse transcriptase sequencing of small-subunit rRNA. Int J Syst Bacteriol. 1991;41(3):347–354. doi:10.1099/00207713-41-3-347. 1715737

[23] Dzink JL, Sheenan MT, Socransky SS. Proposal of three subspecies of Fusobacterium nucleatum knorr 1922: Fusobacterium nucleatum subsp. nucleatum subsp. nov. comb. nov.; Fusobacterium nucleatum subsp. polymorphum subsp. nov. nom. rev. comb. nov.; and Fusobacterium nucleatum subsp. vincentii subsp. nov. nom. rev. comb. nov. Int J Syst Bacteriol. 1990;40(1):74–78. doi:10.1099/00207713-40-1-74. 2223601

[24] Kook J-K, Park S-N, Lim YK, Cho E, Jo E, Roh H, Shin Y, Paek J, Kim H-S, Kim H, et al. Genome-based reclassification of Fusobacterium nucleatum subspecies at the species level. Curr Microbiol. 2017;74(10):1137–1147. doi:10.1007/s00284-017-1296-9. 28687946

[25] Ang MY, Dutta A, Wee WY, Dymock D, Paterson IC, Choo SW. Comparative genome analysis of Fusobacterium nucleatum. Genome Biol Evol. 2016;8(9):2928–2938. doi:10.1093/gbe/evw199. 27540086 PMC5630926

[26] Ma X, Sun T, Zhou J, Zhi M, Shen S, Wang Y, Gu X, Li Z, Gao H, Wang P, et al. Pangenomic study of Fusobacterium nucleatum reveals the distribution of pathogenic genes and functional clusters at the subspecies and strain levels. Microbiol Spectr. 2023;11(3):e0518422. doi:10.1128/spectrum.05184-22. 37042769 PMC10269558

[27] Queen J, Domingue JC, White JR, Stevens C, Udayasuryan B, Nguyen TTD, Wu S, Ding H, Fan H, McMann M, et al. Comparative analysis of colon cancer- derived Fusobacterium nucleatum subspecies: inflammation and colon tumorigenesis in murine models. mBio. 2021;13(1):e0299121. doi:10.1128/mbio.02991-21. 35130731 PMC8822350

[28] Krieger M, AbdelRahman YM, Choi D, Palmer EA, Yoo A, McGuire S, Kreth J, Merritt J. Stratification of Fusobacterium nucleatum by local health status in the oral cavity defines its subspecies disease association. Cell Host & Microbe. 2024;32(4):479–488.e4. doi:10.1016/j.chom.2024.02.010. 38479393 PMC11018276

[29] Weiss C, Mercado DG. Demonstration of type specific proteins in extracts of Fusobacteria. J Exp Med. 1938;67(1):49–59. doi:10.1084/jem.67.1.49. 19870709 PMC2133543

[30] Knorr M. Über die fusospirillare Symbiose, die Gattung Fusobacterium (K.B. Lehmann) und Spirillum sputigenum. Zentralbl für Bakteriologie, Parasitenkd, Infektionskrankh und Hyg, Abteilung. 1922;89:4–22.

[31] Loesche WJ, Gibbons RJ. Amino acid fermentation by Fusobacterium nucleatum. Arch Oral Biol. 1968;13(2):191–IN11. doi:10.1016/0003-9969(68)90051-4. 5238887

[32] Abbott DM, Sudo SZ. Gliding motility and actinomycin D sensitivity of Fusobacterium nucleatum and other gram-negative rods. Infect Immun. 1977;17(3):655–660. doi:10.1128/iai.17.3.655-660.1977. 332633 PMC421178

[33] Falkler WA, Hawley CE. Hemagglutinating activity of fusobacterium nucleatum. Infect Immun. 1977;15(1):230–238. doi:10.1128/iai.15.1.230-238.1977. 401771 PMC421353

[34] Fredriksen G, Hofstad T. Chemotypes of fusobacterium nucleatum lipopolysaccharides. Acta Pathol Microbiol Scand B. 1978;86B(1–6):41–46. doi:10.1111/j.1699-0463.1978.tb00006.x. 665242

[35] Hase S, Hofstad T, Rietschel ET. Chemical structure of the lipid a component of lipopolysaccharides from fusobacterium nucleatum. J Bacteriol. 1977;129(1):9–14. doi:10.1128/jb.129.1.9-14.1977. 830651 PMC234887

[36] Loesche WJ. Oxygen sensitivity of various anaerobic bacteria. Appl Microbiol. 1969;18(5):723–727. doi:10.1128/am.18.5.723-727.1969. 5370458 PMC378078

[37] Mongiello JR, Falkler WA. Sugar inhibition of oral fusobacterium nucleatum haemagglutination and cell binding. Arch Oral Biol. 1979;24(7):539–545. doi:10.1016/0003-9969(79)90133-x. 44185

[38] Slots J. The predominant cultivable microflora of advanced periodontitis. Scand J Dent Res. 1977;85(2):114–121. doi:10.1111/j.1600-0722.1977.tb00541.x. 320648

[39] Sutter VL, Finegold SM. Rosamicin: in vitro activity against anaerobes and comparison with erythromycin. Antimicrob Agents Chemother. 1976;9(2):350–351. doi:10.1128/AAC.9.2.350. 1267432 PMC429526

[40] Tew JG, Thomas SS, Ranney RR. Fusobacterium nucleatum-mediated immunomodulation of the in vitro secondary antibody response to tetanus toxoid and actinobacillus actinomycetemcomitans. J Periodontal Res. 1987;22(6):506–512. doi:10.1111/j.1600-0765.1987.tb02062.x. 2963111

[41] Vasstrand EN, Hofstad T, Endresen C, Jensen HB. Demonstration of lanthionine as a natural constituent of the peptidoglycan of fusobacterium nucleatum. Infect Immun. 1979;25(3):775–780. doi:10.1128/iai.25.3.775-780.1979. 500186 PMC414514

[42] Dzink JL, Socransky SS. Amino acid utilization by fusobacterium nucleatum grown in a chemically defined medium. Oral Microbiol Immunol. 1990;5(3):172–174. doi:10.1111/j.1399-302X.1990.tb00418.x. 2080074

[43] Gharbia SE, Shah HN, Lawson PA, Haapasalo M. The distribution and frequency of fusobacterium nucleatum subspecies in the human oral cavity. Oral Microbiol Immunol. 1990;5(6):324–327. doi:10.1111/j.1399-302x.1990.tb00434.x. 2098710

[44] Xie H, Gibbons RJ, Hay DI. Adhesive properties of strains of fusobacterium nucleatum of the subspecies nucleatum, vincentii and polymorphum. Oral Microbiol Immunol. 1991;6(5):257–263. doi:10.1111/j.1399-302x.1991.tb00488.x. 1820561

[45] Gharbia SE, Shah HN. Comparison of the amino acid uptake profile of reference and clinical isolates of fusobacterium nucleatum subspecies. Oral Microbiol Immunol. 1991;6(5):264–269. doi:10.1111/j.1399-302x.1991.tb00489.x. 1820562

[46] Bennett KW, Eley A. Fusobacteria: new taxonomy and related diseases. J Med Microbiol. 1993;39(4):246–254. doi:10.1099/00222615-39-4-246. 8411084

[47] Morris ML, Andrews RH, Rogers AH. The use of allozyme electrophoresis to assess genetic heterogeneity among previously subspeciated isolates of fusobacterium nucleatum. Oral Microbiol Immunol. 1996;11(1):15–21. doi:10.1111/j.1399-302x.1996.tb00331.x. 8604250

[48] Morris ML, Andrews RH, Rogers AH. Investigations of the taxonomy and systematics of fusobacterium nucleatum using allozyme electrophoresis. Int J Syst Bacteriol. 1997;47(1):103–110. doi:10.1099/00207713-47-1-103. 8995811

[49] Rogers AH. Studies on fusobacteria associated with periodontal diseases. Aust Dent J. 1998;43(2):105–109. doi:10.1111/j.1834-7819.1998.tb06098.x. 9612984

[50] Jousimies-Somer H. Recently described clinically important anaerobic bacteria: taxonomic aspects and update. Clin Infect Dis. 1997;Suppl 25(s2):S78–87. doi:10.1086/516227. 9310640

[51] George KS, Reynolds MA, Falkler WA. Arbitrarily primed polymerase chain reaction fingerprinting and clonal analysis of oral fusobacterium nucleatum isolates. Oral Microbiol Immunol. 1997;12(4):219–226. doi:10.1111/j.1399-302x.1997.tb00382.x. 9467390

[52] Socransky SS, Haffajee AD, Cugini MA, Smith C, Kent RL. Microbial complexes in subgingival plaque. J Clin Periodontol. 1998;25(2):134–144. doi:10.1111/j.1600-051x.1998.tb02419.x. 9495612

[53] Kapatral V, Anderson I, Ivanova N, Reznik G, Los T, Lykidis A, Bhattacharyya A, Bartman A, Gardner W, Grechkin G, et al. Genome sequence and analysis of the oral bacterium fusobacterium nucleatum strain ATCC 25586. J Bacteriol. 2002;184(7):2005–2018. doi:10.1128/JB.184.7.2005-2018.2002. 11889109 PMC134920

[54] Kapatral V, Ivanova N, Anderson I, Reznik G, Bhattacharyya A, Gardner WL, Mikhailova N, Lapidus A, Larsen N, D’Souza M, et al. Genome analysis of F. nucleatum sub spp vincentii and its comparison with the genome of F. nucleatum ATCC 25586. Genome Res. 2003;13(6a):1180–1189. doi:10.1101/gr.566003. 12799352 PMC403646

[55] Olsen I, Shah HN. International committee on systematics of prokaryotes subcommittee on the taxonomy of gram-negative anaerobic rods. Int J Systematic Evol Microbiol. 2003;53(3):923–924. doi:10.1099/ijs.0.02612-0.

[56] Park S-N, Kong S-W, Park M-S, Lee J-W, Cho E, Lim YK, Choi M-H, Kim H-S, Chang Y-H, Shin JH, et al. Draft genome sequence of fusobacterium nucleatum subsp. fusiforme ATCC 51190 T. J Bacteriol. 2012;194(19):5445–5446. doi:10.1128/JB.01138-12. 22965077 PMC3457189

[57] Park S-N, Cho E, Kim H-S, Kim D-S, Jung J, Baek J-H, Kyong Lim Y, Jo E, Chang Y-H, Hwan Shin J, et al. Draft genome sequence of fusobacterium nucleatum subsp. animalis ChDC F324, isolated from a human subgingival plaque in the Republic of Korea. Genome Announc. 2013;1(6):e01042–13. doi:10.1128/genomeA.01042-13. 24336380 PMC3861433

[58] Kook J-K, Kim M-K, Seong J-H, Kim D-K, Kim B-O, Park J-C, Kim K-K, Choe S-J, Min B-M. A new method for rapid screening of bacterial species- or subspecies-specific DNA probes. FEMS Microbiol Lett. 2003;219(1):121–127. doi:10.1016/S0378-1097(03)00021-1. 12594033

[59] Woese CR, Stackebrandt E, Macke TJ, Fox GE. A phylogenetic definition of the major eubacterial taxa. Syst Appl Microbiol. 1985;6(2):143–151. doi:10.1016/s0723-2020(85)80047-3. 11542017

[60] Böttger EC. Rapid determination of bacterial ribosomal RNA sequences by direct sequencing of enzymatically amplified DNA. FEMS Microbiol Lett. 1989;53(1–2):171–176. doi:10.1111/j.1574-6968.1989.tb03617.x. 2482222

[61] Clarridge JE. Impact of 16S rRNA gene sequence analysis for identification of bacteria on clinical microbiology and infectious diseases. Clin Microbiol Rev. 2004;17(4):840–862. doi:10.1128/CMR.17.4.840-862.2004. 15489351 PMC523561

[62] Kim H-S, Lee D-S, Chang Y-H, Kim MJ, Koh S, Kim J, Seong J-H, Song SK, Shin HS, Son J-B, et al. Application of rpoB and zinc protease gene for use in molecular discrimination of fusobacterium nucleatum subspecies. J Clin Microbiol. 2010;48(2):545–553. doi:10.1128/JCM.01631-09. 19955278 PMC2815611

[63] Conrads G, Claros MC, Citron DM, Tyrrell KL, Merriam V, Goldstein EJC. 16S-23S rDNA internal transcribed spacer sequences for analysis of the phylogenetic relationships among species of the genus fusobacterium. Int J Syst Evol Microbiol. 2002;52(2):493–499. doi:10.1099/00207713-52-2-493. 11931161

[64] Citron DM. Update on the taxonomy and clinical aspects of the genus fusobacterium. Clin Infect Dis. 2002;35(s1):S22–S27. doi:10.1086/341916. 12173104

[65] Goldstein EJ, Citron DM, Merriam CV, Warren Y, Tyrrell K. Comparative in vitro activities of GAR-936 against aerobic and anaerobic animal and human bite wound pathogens. Antimicrob Agents Chemother. 2000;44:2747–2751. doi:10.1128/AAC.44.10.2747-2751.2000. 10991855 PMC90146

[66] Conrads G, Citron DM, Mutters R, Jang S, Goldstein EJC. Fusobacterium canifelinum sp. nov. from the oral cavity of cats and dogs. Syst Appl Microbiol. 2004;27(4):407–413. doi:10.1078/0723202041438509. 15368845

[67] Dahlén G, Charalampakis G, Abrahamsson I, Bengtsson L, Falsen E. Predominant bacterial species in subgingival plaque in dogs. J Periodontal Res. 2012;47(3):354–364. doi:10.1111/j.1600-0765.2011.01440.x. 22181039

[68] Richardson M, Ren J, Rubinstein MR, Taylor JA, Friedman RA, Shen B, Han YW. Analysis of 16S rRNA genes reveals reduced fusobacterial community diversity when translocating from saliva to GI sites. Gut Microbes. 2020;12(1):1814120. doi:10.1080/19490976.2020.1814120. 33054632 PMC7577115

[69] Nardello LCL, Amado PPP, Franco DC, Cazares RXR, Nogales CG, Mayer MPA, Karygianni L, Thurnheer T, Pinheiro ET. Next-generation sequencing to assess potentially active bacteria in endodontic infections. J Endod. 2020;46(8):1105–1112. doi:10.1016/j.joen.2020.05.004. 32497654

[70] Strauss J, White A, Ambrose C, McDonald J, Allen-Vercoe E. Phenotypic and genotypic analyses of clinical fusobacterium nucleatum and fusobacterium periodonticum isolates from the human gut. Anaerobe. 2008;14(6):301–309. doi:10.1016/j.anaerobe.2008.12.003. 19114111

[71] Bi D, Zhu Y, Gao Y, Li H, Zhu X, Wei R, Xie R, Wei Q, Qin H. A newly developed pcr-based method revealed distinct fusobacterium nucleatum subspecies infection patterns in colorectal cancer. Microb Biotechnol. 2021;14(5):2176–2186. doi:10.1111/1751-7915.13900. 34309194 PMC8449656

[72] Sanders BE, Umana A, Lemkul JA, Slade DJ. Erratum for Sanders et al. “FusoPortal: an interactive repository of hybrid MinION-sequenced fusobacterium genomes improves gene identification and characterization”. mSphere. 2018;3(4):e00228–18. doi:10.1128/mSphere.00379-18. 29976644 PMC6034081

[73] Ponath F, Tawk C, Zhu Y, Barquist L, Faber F, Vogel J. RNA landscape of the emerging cancer-associated microbe fusobacterium nucleatum. Nat Microbiol. 2021;6(8):1007–1020. doi:10.1038/s41564-021-00927-7. 34239075

[74] Nie S, Tian B, Wang X, Pincus DH, Welker M, Gilhuley K, Lu X, Han YW, Tang Y-W. Fusobacterium nucleatum subspecies identification by matrix-assisted laser desorption ionization–time of flight Mass spectrometry. J Clin Microbiol. 2015;53(4):1399–1402. doi:10.1128/JCM.00239-15. 25653408 PMC4365237

[75] Morsi H, Golizeh M, Brosseau N, Janati AI, Emami E, Ndao M, Tran SD. Detection of fusobacterium nucleatum subspecies in the saliva of pre-colorectal cancer patients, using tandem mass spectrometry. Arch Oral Biol. 2022;134:105337. doi:10.1016/j.archoralbio.2021.105337. 34929558

[76] Gmür R, Munson MA, Wade WG. Genotypic and phenotypic characterization of fusobacteria from Chinese and European patients with inflammatory periodontal diseases. Syst Appl Microbiol. 2006;29(2):120–130. doi:10.1016/j.syapm.2005.07.011. 16464693

[77] Manson McGuire A, Cochrane K, Griggs AD, Haas BJ, Abeel T, Zeng Q, Nice JB, MacDonald H, Birren BW, Berger BW, et al. Evolution of invasion in a diverse set of fusobacterium species. mBio. 2014;5(6):e01864. doi:10.1128/mBio.01864-14. 25370491 PMC4222103

[78] Richter M, Rosselló-Móra R. Shifting the genomic gold standard for the prokaryotic species definition. Proc Natl Acad Sci USA. 2009;106(45):19126–19131. doi:10.1073/pnas.0906412106. 19855009 PMC2776425

[79] Zhou P, Gc B, Stolte F, Wu C. Use of CRISPR interference for efficient and rapid gene inactivation in Fusobacterium nucleatum. Appl Environ Microbiol. 2024;90(2):e01665–23. doi:10.1128/aem.01665-23. 38185820 PMC10880640

[80] Sanders BE, Umaña A, Nguyen TTD, Williams KJ, Yoo CC, Casasanta MA, Wozniak B, Slade DJ. Type IV pili facilitated natural competence in Fusobacterium nucleatum. Anaerobe. 2023;82:102760. doi:10.1016/j.anaerobe.2023.102760. 37451427

[81] Merritt J, Kreth J. Illuminating the oral microbiome and its host interactions: tools and approaches for molecular microbiology studies. FEMS Microbiol Rev. 2023;47(6):fuac050. doi:10.1093/femsre/fuac050. 36549660 PMC10719069

[82] Han YW, Ikegami A, Rajanna C, Kawsar HI, Zhou Y, Li M, Sojar HT, Genco RJ, Kuramitsu HK, Deng CX. Identification and characterization of a novel adhesin unique to oral fusobacteria. J Bacteriol. 2005;187(15):5330–5340. doi:10.1128/jb.187.15.5330-5340.2005. 16030227 PMC1196005

[83] Han YW, Ikegami A, Chung P, Zhang L, Deng CX. Sonoporation is an efficient tool for intracellular fluorescent dextran delivery and one-step double-crossover mutant construction in Fusobacterium nucleatum. Appl Environ Microbiol. 2007;73(11):3677–3683. doi:10.1128/AEM.00428-07. 17449701 PMC1932673

[84] Ikegami A, Chung P, Han YW. Complementation of the fadA mutation in Fusobacterium nucleatum demonstrates that the surface-exposed adhesin promotes cellular invasion and placental colonization. Infect Immun. 2009;77(7):3075–3079. doi:10.1128/IAI.00209-09. 19398541 PMC2708594

[85] Haake SK, Yoder SC, Attarian G, Podkaminer K. Native plasmids of fusobacterium nucleatum: characterization and use in development of genetic systems. J Bacteriol. 2000;182(4):1176–1180. doi:10.1128/jb.182.4.1176-1180.2000. 10648549 PMC94399

[86] Haake SK, Yoder S, Gerardo SH. Efficient gene transfer and targeted mutagenesis in fusobacterium nucleatum. Plasmid. 2006;55(1):27–38. doi:10.1016/j.plasmid.2005.06.002. 16115683 PMC1592470

[87] Kaplan CW, Ma X, Paranjpe A, Jewett A, Lux R, Kinder-Haake S, Shi W. Fusobacterium nucleatum outer membrane proteins Fap2 and RadD induce cell death in human lymphocytes. Infect Immun. 2010;78(11):4773–4778. doi:10.1128/IAI.00567-10. 20823215 PMC2976331

[88] Umaña A, Nguyen TTD, Sanders BE, Williams KJ, Wozniak B, Slade DJ, Bondy-Denomy J. Enhanced fusobacterium nucleatum genetics using host DNA methyltransferases to bypass restriction-modification systems. J Bacteriol. 2022;204(12):e0027922. doi:10.1128/jb.00279-22. 36326270 PMC9764991

[89] Rickard AH, Gilbert P, High NJ, Kolenbrander PE, Handley PS. Bacterial coaggregation: an integral process in the development of multi-species biofilms. Trends Microbiol. 2003;11(2):94–100. doi:10.1016/s0966-842x(02)00034-3. 12598132

[90] Kolenbrander PE, Palmer RJ, Periasamy S, Jakubovics NS. Oral multispecies biofilm development and the key role of cell–cell distance. Nat Rev Microbiol. 2010;8(7):471–480. doi:10.1038/nrmicro2381. 20514044

[91] Kolenbrander PE, Andersen RN, Moore LV. Coaggregation of fusobacterium nucleatum, selenomonas flueggei, selenomonas infelix, selenomonas noxia, and Selenomonas sputigena with strains from 11 genera of oral bacteria. Infect Immun. 1989;57(10):3194–3203. doi:10.1128/iai.57.10.3194-3203.1989. 2777378 PMC260789

[92] Andersen RN, Ganeshkumar N, Kolenbrander PE. Helicobacter pylori adheres selectively to fusobacterium spp. Oral Microbiol Immunol. 1998;13(1):51–54. doi:10.1111/j.1399-302x.1998.tb00751.x. 9573823

[93] Kinder SA, Holt SC. Characterization of coaggregation between Bacteroides gingivalis T22 and fusobacterium nucleatum T18. Infect Immun. 1989;57(11):3425–3433. doi:10.1128/iai.57.11.3425-3433.1989. 2478473 PMC259844

[94] Kolenbrander PE, Parrish KD, Andersen RN, Greenberg EP. Intergeneric coaggregation of oral Treponema spp. With fusobacterium spp. And intrageneric coaggregation among fusobacterium spp. Infect Immun. 1995;63(12):4584–4588. doi:10.1128/iai.63.12.4584-4588.1995. 7591109 PMC173658

[95] Kinder SA, Holt SC. Localization of the fusobacterium nucleatum T18 adhesin activity mediating coaggregation with porphyromonas gingivalis T22. J Bacteriol. 1993;175(3):840–850. doi:10.1128/jb.175.3.840-850.1993. 8380804 PMC196226

[96] Horiuchi A, Kokubu E, Warita T, Ishihara K. Synergistic biofilm formation by Parvimonas micra and fusobacterium nucleatum. Anaerobe. 2020;62:102100. doi:10.1016/j.anaerobe.2019.102100. 31521732

[97] Li Q, Wang H, Tan L, Zhang S, Lin L, Tang X, Pan Y. Oral pathogen fusobacterium nucleatum coaggregates with Pseudomonas aeruginosa to modulate the inflammatory cytotoxicity of pulmonary epithelial cells. Front Cell Infect Microbiol. 2021;11:643913. doi:10.3389/fcimb.2021.643913. 33816348 PMC8017200

[98] Roques CG, El Kaddouri S, Barthet P, Duffort JF, Arellano M. Fusobacterium nucleatum involvement in adult periodontitis and possible modification of strain classification. J Periodontol. 2000;71(7):1144–1150. doi:10.1902/jop.2000.71.7.1144. 10960022

[99] Yang R, Liu T, Pang C, Cai Y, Lin Z, Guo L, Wei X. The regulatory effect of coaggregation between fusobacterium nucleatum and streptococcus gordonii on the synergistic virulence to human gingival epithelial cells. Front Cell Infect Microbiol. 2022;12:879423. doi:10.3389/fcimb.2022.879423. 35573793 PMC9100429

[100] Robinson AV, Allen-Vercoe E. Strain specificity in fusobacterial co-aggregation with colorectal cancer-relevant species. Anaerobe. 2023;82:102758. doi:10.1016/j.anaerobe.2023.102758. 37423597

[101] Muchova M, Balacco DL, Grant MM, Chapple ILC, Kuehne SA, Hirschfeld J. Fusobacterium nucleatum subspecies differ in biofilm forming ability in vitro. Front Oral Health. 2022;3:853618. doi:10.3389/froh.2022.853618. 35368312 PMC8967363

[102] Meng Q, Gao Q, Mehrazarin S, Tangwanichgapong K, Wang Y, Huang Y, Pan Y, Robinson S, Liu Z, Zangiabadi A, et al. Fusobacterium nucleatum secretes amyloid-like FadA to enhance pathogenicity. EMBO Rep. 2021;22(7):e52891. doi:10.15252/embr.202152891. 34184813 PMC8406402

[103] Thurnheer T, Karygianni L, Flury M, Belibasakis GN. Fusobacterium species and subspecies differentially affect the composition and architecture of supra- and subgingival biofilms models. Front Microbiol. 2019. doi:10.3389/fmicb.2019.01716. PMC6683768 31417514

[104] Shi S, Liu Y, Wang Z, Jin X, Yan W, Guo X, Lin B, Wang H, Li B, Zheng J, et al. Fusobacterium nucleatum induces colon anastomosis leak by activating epithelial cells to express MMP9. Front Microbiol. 2022;13:1031882. doi:10.3389/fmicb.2022.1031882. 36590433 PMC9794562

[105] Sun J, Tang Q, Yu S, Xie M, Zheng W, Chen G, Yin Y, Huang X, Wo K, Lei H, et al. F. nucleatum facilitates oral squamous cell carcinoma progression via GLUT1-driven lactate production. eBiomedicine 88. eBiomedicine. 2023;88:104444. doi:10.1016/j.ebiom.2023.104444. 36709580 PMC9900488

[106] Parhi L, Alon-Maimon T, Sol A, Nejman D, Shhadeh A, Fainsod-Levi T, Yajuk O, Isaacson B, Abed J, Maalouf N, et al. Breast cancer colonization by fusobacterium nucleatum accelerates tumor growth and metastatic progression. Nat Commun. 2020;11(1):3259. doi:10.1038/s41467-020-16967-2. 32591509 PMC7320135

[107] Gao Y, Bi D, Xie R, Li M, Guo J, Liu H, Guo X, Fang J, Ding T, Zhu H, et al. Correction to: fusobacterium nucleatum enhances the efficacy of PD-L1 blockade in colorectal cancer. Sig Transduct Target Ther. 2021;6(1):1–10. doi:10.1038/s41392-021-00840-9. PMC8602417 34795206

[108] Liu Y, Baba Y, Ishimoto T, Tsutsuki H, Zhang T, Nomoto D, Okadome K, Yamamura K, Harada K, Eto K, et al. Fusobacterium nucleatum confers chemoresistance by modulating autophagy in oesophageal squamous cell carcinoma. Br J Cancer. 2021;124(5):963–974. doi:10.1038/s41416-020-01198-5. 33299132 PMC7921654

[109] Nomoto D, Baba Y, Liu Y, Tsutsuki H, Okadome K, Harada K, Ishimoto T, Iwatsuki M, Iwagami S, Miyamoto Y, et al. Fusobacterium nucleatum promotes esophageal squamous cell carcinoma progression via the NOD1/RIPK2/NF-κB pathway. Cancer Lett. 2022;530:59–67. doi:10.1016/j.canlet.2022.01.014. 35033591

[110] Rubinstein MR, Baik JE, Lagana SM, Han RP, Raab WJ, Sahoo D, Dalerba P, Wang TC, Han YW. Fusobacterium nucleatum promotes colorectal cancer by inducing Wnt/β-catenin modulator annexin A1. EMBO Rep. 2019;20(4):e47638. doi:10.15252/embr.201847638. 30833345 PMC6446206

[111] Wu Y, Guo S, Chen F, Li Y, Huang Y, Liu W, Zhang G. Fn-dps, a novel virulence factor of fusobacterium nucleatum, disrupts erythrocytes and promotes metastasis in colorectal cancer. PLOS Pathog. 2023;19(1):e1011096. doi:10.1371/journal.ppat.1011096. 36693067 PMC9873182

[112] Gu X, Song L, Li L, Liu T, Zhang M, Li Z, Wang P, Li M, Zuo X. Fusobacterium nucleatum causes microbial dysbiosis and exacerbates visceral hypersensitivity in a colonization-independent manner. Front Microbiol. 2020;11. doi:10.3389/fmicb.2020.01281. PMC7358639 32733392

[113] Fardini Y, Wang X, Témoin S, Nithianantham S, Lee D, Shoham M, Han YW. Fusobacterium nucleatum adhesin FadA binds vascular endothelial cadherin and alters endothelial integrity. Mol Microbiol. 2011;82(6):1468–1480. doi:10.1111/j.1365-2958.2011.07905.x. 22040113 PMC3237733

[114] Casasanta MA, Yoo CC, Udayasuryan B, Sanders BE, Umaña A, Zhang Y, Peng H, Duncan AJ, Wang Y, Li L, et al. Fusobacterium nucleatum host-cell binding and invasion induces IL-8 and CXCL1 secretion that drives colorectal cancer cell migration. Sci Signal. 2020;13(641):eaba9157. doi:10.1126/scisignal.aba9157. 32694172 PMC7454160

[115] Fardini Y, Chung P, Dumm R, Joshi N, Han YW. Transmission of diverse oral bacteria to murine placenta: evidence for the oral microbiome as a potential source of intrauterine infection. Infect Immun. 2010;78(4):1789–1796. doi:10.1128/IAI.01395-09. 20123706 PMC2849412

[116] Kurgan Ş, Kansal S, Nguyen D, Stephens D, Koroneos Y, Hasturk H, Van Dyke TE, Kantarci A. Strain-specific impact of fusobacterium nucleatum on neutrophil function. J Periodontol. 2017;88(4):380–389. doi:10.1902/jop.2016.160212. 27762731

[117] Liu H, Redline RW, Han YW. Fusobacterium nucleatum induces fetal death in mice via stimulation of TLR4-mediated placental inflammatory response. J Immunol. 2007;179(4):2501–2508. doi:10.4049/jimmunol.179.4.2501. 17675512

[118] Sveen K, Skaug N. Bone resorption stimulated by lipopolysaccharides from bacteroides, fusobacterium and Veillonella, and by the lipid a and the polysaccharide part of fusobacterium lipopolysaccharide. Scand J Dent Res. 1980;88(6):535–542. doi:10.1111/j.1600-0722.1980.tb01264.x. 7017892

[119] Johnson L, Almeida-da-Silva CLC, Takiya CM, Figliuolo V, Rocha GM, Weissmüller G, Scharfstein J, Coutinho-Silva R, Ojcius DM. Oral infection of mice with Fusobacterium nucleatum results in macrophage recruitment to the dental pulp and bone resorption. Biomed J. 2018;41(3):184–193. doi:10.1016/j.bj.2018.05.001. 30080658 PMC6138822

[120] Doron L, Coppenhagen-Glazer S, Ibrahim Y, Eini A, Naor R, Rosen G, Bachrach G. Identification and characterization of fusolisin, the Fusobacterium nucleatum autotransporter serine protease. PLoS One. 2014;9(10):e111329. doi:10.1371/journal.pone.0111329. 25357190 PMC4214739

[121] Mahtout H, Chandad F, Rojo JM, Grenier D. Fusobacterium nucleatum binding to complement regulatory protein CD46 modulates the expression and secretion of cytokines and matrix metalloproteinases by oral epithelial cells. J Periodontol. 2011;82(2):311–319. doi:10.1902/jop.2010.100458. 20843232

[122] Chen Y, Huang Z, Tang Z, Huang Y, Huang M, Liu H, Ziebolz D, Schmalz G, Jia B, Zhao J. More than just a periodontal pathogen –the research progress on Fusobacterium nucleatum. Front Cell Infect Microbiol. 2022;12. doi:10.3389/fcimb.2022.815318. PMC8851061 35186795

[123] Chen Y, Shi T, Li Y, Huang L, Yin D. Fusobacterium nucleatum: the opportunistic pathogen of periodontal and peri-implant diseases. Front Microbiol. 2022;13:860149. doi:10.3389/fmicb.2022.860149. 35369522 PMC8966671

[124] George N, Flamiatos E, Kawasaki K, Kim N, Carriere C, Phan B, Joseph R, Strauss S, Kohli R, Choi D, et al. Oral microbiota species in acute apical endodontic abscesses. J Oral Microbiol. 2016;8(1):30989. doi:10.3402/jom.v8.30989. 26983837 PMC4794734

[125] Saar M, Vaikjärv R, Parm Ü, Kasenõmm P, Kõljalg S, Sepp E, Jaagura M, Salumets A, Štšepetova J, Mändar R. Unveiling the etiology of peritonsillar abscess using next generation sequencing. Ann Clin Microbiol Antimicrobials. 2023;22(1):98. doi:10.1186/s12941-023-00649-0. PMC10633907 37940951

[126] Carda-Diéguez M, Rosier BT, Lloret S, Llena C, Mira A. The tongue biofilm metatranscriptome identifies metabolic pathways associated with the presence or absence of halitosis. NPJ Biofilms Microbiomes. 2022;8(1):100. doi:10.1038/s41522-022-00364-2. 36535943 PMC9763428

[127] Pietrobon G, Tagliabue M, Stringa LM, De Berardinis R, Chu F, Zocchi J, Carlotto E, Chiocca S, Ansarin M. Leukoplakia in the oral cavity and oral microbiota: a comprehensive review. Cancers (Basel). 2021;13(17):4439. doi:10.3390/cancers13174439. 34503249 PMC8431082

[128] Zhou L, Mao H-Q, Li J-Q, Chen Z, Zhang L. Fusobacterium nucleatum exacerbates the progression of pulpitis by regulating the sting-dependent pathway. Faseb J. 2024;38(1):e23357. doi:10.1096/fj.202301648R. 38085169

[129] Song B, Xian W, Sun Y, Gou L, Guo Q, Zhou X, Ren B, Cheng L. Akkermansia muciniphila inhibited the periodontitis caused by Fusobacterium nucleatum. NPJ Biofilms Microbiomes. 2023;9(1):49. doi:10.1038/s41522-023-00417-0. 37460552 PMC10352368

[130] Kageyama S, Takeshita T, Asakawa M, Shibata Y, Takeuchi K, Yamanaka W, Yamashita Y. Relative abundance of total subgingival plaque-specific bacteria in salivary microbiota reflects the overall periodontal condition in patients with periodontitis. PLOS ONE. 2017;12(4):e0174782. doi:10.1371/journal.pone.0174782. 28369125 PMC5378373

[131] Chaushu S, Wilensky A, Gur C, Shapira L, Elboim M, Halftek G, Polak D, Achdout H, Bachrach G, Mandelboim O. Direct recognition of Fusobacterium nucleatum by the NK cell natural cytotoxicity receptor NKp46 aggravates periodontal disease. PLOS Pathog. 2012;8(3):e1002601. doi:10.1371/journal.ppat.1002601. 22457623 PMC3310798

[132] Liu P-F, Haake SK, Gallo RL, Huang C-M. A novel vaccine targeting Fusobacterium nucleatum against abscesses and halitosis. Vaccine. 2009;27(10):1589–1595. doi:10.1016/j.vaccine.2008.12.058. 19162109 PMC3057132

[133] Zhang W, Chen Y, Shi Q, Hou B, Yang Q. Identification of bacteria associated with periapical abscesses of primary teeth by sequence analysis of 16S rDNA clone libraries. Microb Pathog. 2020;141:103954. doi:10.1016/j.micpath.2019.103954. 31891793

[134] Basic A, Blomqvist M, Dahlén G, Svensäter G. The proteins of Fusobacterium spp. involved in hydrogen sulfide production from L-cysteine. BMC Microbiol. 2017;17(1):61. doi:10.1186/s12866-017-0967-9. 28288582 PMC5348791

[135] Amer A, Galvin S, Healy CM, Moran GP. The microbiome of potentially malignant oral leukoplakia exhibits enrichment for Fusobacterium, Leptotrichia, campylobacter, and rothia species. Front Microbiol. 2017;8:2391. doi:10.3389/fmicb.2017.02391. 29250055 PMC5717034

[136] Walter J, Margosch D, Hammes WP, Hertel C. Detection of Fusobacterium species in human feces using genus-specific PCR primers and denaturing gradient gel electrophoresis. Microb Ecol Health Dis. 2002;14(3):129–132. doi:10.1080/089106002320644294.

[137] Hill GB. Preterm birth: associations with genital and possibly oral microflora. Ann Periodontol. 1998;3(1):222–232. doi:10.1902/annals.1998.3.1.222. 9722706

[138] Han YW, Wang X. Mobile microbiome: oral bacteria in extra-oral infections and inflammation. J Dent Res. 2013;92(6):485–491. doi:10.1177/0022034513487559. 23625375 PMC3654760

[139] Wang X, Buhimschi CS, Temoin S, Bhandari V, Han YW, Buhimschi IA. Comparative microbial analysis of paired amniotic fluid and cord blood from pregnancies complicated by preterm birth and early-onset neonatal sepsis. PLOS ONE. 2013;8(2):e56131. doi:10.1371/journal.pone.0056131. 23437088 PMC3577789

[140] Vander Haar EL, Wu G, Gyamfi-Bannerman C, Thomas C, Wapner RJ, Reddy UM, Zhao L, Silver RM, Goldenberg RL, Han YW. Microbial analysis of umbilical cord blood reveals novel pathogens associated with stillbirth and early preterm birth. mBio. 2022;13(5):e02036–22. doi:10.1128/mbio.02036-22. 35993728 PMC9600380

[141] Han YW, Fardini Y, Chen C, Iacampo KG, Peraino VA, Shamonki JM, Redline RW. Term stillbirth caused by oral fusobacterium nucleatum. Obstet Gynecol. 2010;115(2):442–445. doi:10.1097/AOG.0b013e3181cb9955. 20093874 PMC3004155

[142] Gonzales-Marin C, Spratt DA, Allaker RP. Maternal oral origin of Fusobacterium nucleatum in adverse pregnancy outcomes as determined using the 16S–23S rRNA gene intergenic transcribed spacer region. J Med Microbiol. 2013;62(1):133–144. doi:10.1099/jmm.0.049452-0. 23002071

[143] Vander Haar EL, So J, Gyamfi-Bannerman C, Han YW. Fusobacterium nucleatum and adverse pregnancy outcomes: epidemiological and mechanistic evidence. Anaerobe. 2018;50:55–59. doi:10.1016/j.anaerobe.2018.01.008. 29409815 PMC6750227

[144] Stockham S, Stamford JE, Roberts CT, Fitzsimmons TR, Marchant C, Bartold PM, Zilm PS. Abnormal pregnancy outcomes in mice using an induced periodontitis model and the haematogenous migration of Fusobacterium nucleatum sub-species to the murine placenta. PLOS ONE. 2015;10(3):e0120050. doi:10.1371/journal.pone.0120050. 25806806 PMC4373690

[145] Venkatesan P. Bacterial infection linked to endometriosis. Lancet Microbe. 2023;4(10):e768. doi:10.1016/S2666-5247(23)00221-5. 37478882

[146] Muraoka A, Suzuki M, Hamaguchi T, Watanabe S, Iijima K, Murofushi Y, Shinjo K, Osuka S, Hariyama Y, Ito M, et al. Fusobacterium infection facilitates the development of endometriosis through the phenotypic transition of endometrial fibroblasts. Sci Transl Med. 2023;15(700):eadd1531. doi:10.1126/scitranslmed.add1531. 37315109

[147] Castellarin M, Warren RL, Freeman JD, Dreolini L, Krzywinski M, Strauss J, Barnes R, Watson P, Allen-Vercoe E, Moore RA, et al. Fusobacterium nucleatum infection is prevalent in human colorectal carcinoma. Genome Res. 2012;22(2):299–306. doi:10.1101/gr.126516.111. 22009989 PMC3266037

[148] Chen H, Jiao J, Wei M, Jiang X, Yang R, Yu X, Zhang G, Zhou X. Metagenomic analysis of the interaction between the gut microbiota and colorectal cancer: a paired-sample study based on the GMrepo database. Gut Pathog. 2022;14(1):48. doi:10.1186/s13099-022-00527-8. 36564826 PMC9784093

[149] Flanagan L, Schmid J, Ebert M, Soucek P, Kunicka T, Liska V, Bruha J, Neary P, Dezeeuw N, Tommasino M, et al. Fusobacterium nucleatum associates with stages of colorectal neoplasia development, colorectal cancer and disease outcome. Eur J Clin Microbiol Infect Dis. 2014;33(8):1381–1390. doi:10.1007/s10096-014-2081-3. 24599709

[150] Hsieh Y-Y, Kuo W-L, Hsu W-T, Tung S-Y, Li C. Fusobacterium nucleatum-induced tumor mutation burden predicts poor survival of gastric cancer patients. Cancers (Basel). 2022;15(1):269. doi:10.3390/cancers15010269. 36612265 PMC9818776

[151] Mehta RS, Nishihara R, Cao Y, Song M, Mima K, Qian ZR, Nowak JA, Kosumi K, Hamada T, Masugi Y, et al. Association of dietary patterns with risk of colorectal cancer subtypes classified by fusobacterium nucleatum in tumor tissue. JAMA Oncol. 2017;3(7):921–927. doi:10.1001/jamaoncol.2016.6374. 28125762 PMC5502000

[152] Mima K, Nishihara R, Qian ZR, Cao Y, Sukawa Y, Nowak JA, Yang J, Dou R, Masugi Y, Song M, et al. Fusobacterium nucleatum in colorectal carcinoma tissue and patient prognosis. Gut. 2016;65(12):1973–1980. doi:10.1136/gutjnl-2015-310101. 26311717 PMC4769120

[153] Shuwen H, Yinhang W, Xingming Z, Jing Z, Jinxin L, Wei W, Kefeng D. Using whole-genome sequencing (WGS) to plot colorectal cancer-related gut microbiota in a population with varied geography. Gut Pathog. 2022;14(1):50. doi:10.1186/s13099-022-00524-x. 36578080 PMC9795735

[154] Wang N, Fang J-Y. Fusobacterium nucleatum, a key pathogenic factor and microbial biomarker for colorectal cancer. Trends Microbiol. 2023;31(2):159–172. doi:10.1016/j.tim.2022.08.010. 36058786

[155] Qin Y, Tong X, Mei W-J, Cheng Y, Zou Y, Han K, Yu J, Jie Z, Zhang T, Zhu S, et al. Consistent signatures in the human gut microbiome of old- and young-onset colorectal cancer. Nat Commun. 2024;15(1):3396. doi:10.1038/s41467-024-47523-x. 38649355 PMC11035630

[156] Senthakumaran T, Moen AEF, Tannæs TM, Endres A, Brackmann SA, Rounge TB, Bemanian V, Tunsjø HS. Microbial dynamics with CRC progression: a study of the mucosal microbiota at multiple sites in cancers, adenomatous polyps, and healthy controls. Eur J Clin Microbiol Infect Dis. 2023;42(3):305–322. doi:10.1007/s10096-023-04551-7. 36703031 PMC9899194

[157] Xu C, Fan L, Lin Y, Shen W, Qi Y, Zhang Y, Chen Z, Wang L, Long Y, Hou T, et al. Fusobacterium nucleatum promotes colorectal cancer metastasis through miR-1322/CCL20 axis and M2 polarization. Gut Microbes. 2021;13(1):1980347. doi:10.1080/19490976.2021.1980347. 34632963 PMC8510564

[158] Rubinstein MR, Wang X, Liu W, Hao Y, Cai G, Han YW. Fusobacterium nucleatum promotes colorectal carcinogenesis by modulating E-cadherin/β-catenin signaling via its FadA adhesin. Cell Host & Microbe. 2013;14(2):195–206. doi:10.1016/j.chom.2013.07.012. 23954158 PMC3770529

[159] Meng Q, Gao Q, Mehrazarin S, Tangwanichgapong K, Wang Y, Huang Y, Pan Y, Robinson S, Liu Z, Zangiabadi A, et al. Fusobacterium nucleatum secretes amyloid‐like FadA to enhance pathogenicity. EMBO Rep. 2021;22(7):e52891. doi:10.15252/embr.202152891. 34184813 PMC8406402

[160] Alon‐Maimon T, Mandelboim O, Bachrach G. Fusobacterium nucleatum and cancer. Periodontol 2000. 2022;89(1):166–180. doi:10.1111/prd.12426. 35244982 PMC9315032

[161] Bronzato JD, Bomfim RA, Edwards DH, Crouch D, Hector MP, Gomes BPFA. Detection of Fusobacterium in oral and head and neck cancer samples: a systematic review and meta-analysis. Archiv Oral Biol. 2020;112:104669. doi:10.1016/j.archoralbio.2020.104669. 32028171

[162] Udayasuryan B, Ahmad RN, Nguyen TTD, Umaña A, Roberts LM, Sobol P, Jones SD, Munson JM, Slade DJ, Verbridge SS. Fusobacterium nucleatum induces proliferation and migration in pancreatic cancer cells through host autocrine and paracrine signaling. Sci Signal. 2022;15(756):eabn4948. doi:10.1126/scisignal.abn4948. 36256708 PMC9732933

[163] Mitsuhashi K, Nosho K, Sukawa Y, Matsunaga Y, Ito M, Kurihara H, Kanno S, Igarashi H, Naito T, Adachi Y, et al. Association of Fusobacterium species in pancreatic cancer tissues with molecular features and prognosis. Oncotarget. 2015;6(9):7209–7220. doi:10.18632/oncotarget.3109. 25797243 PMC4466679

[164] Gaiser RA, Halimi A, Alkharaan H, Lu L, Davanian H, Healy K, Hugerth LW, Ateeb Z, Valente R, Fernández Moro C, et al. Enrichment of oral microbiota in early cystic precursors to invasive pancreatic cancer. Gut. 2019;68(12):2186–2194. doi:10.1136/gutjnl-2018-317458. 30872392 PMC6872446

[165] Hara Y, Baba Y, Oda E, Harada K, Yamashita K, Toihata T, Kosumi K, Iwatsuki M, Miyamoto Y, Tsutsuki H, et al. Presence of Fusobacterium nucleatum in relation to patient survival and an acidic environment in oesophagogastric junction and gastric cancers. Br J Cancer. 2024;131(5):797–807. doi:10.1038/s41416-024-02753-0. 38992099 PMC11368944

[166] Yamamura K, Baba Y, Nakagawa S, Mima K, Miyake K, Nakamura K, Sawayama H, Kinoshita K, Ishimoto T, Iwatsuki M, et al. Human microbiome Fusobacterium nucleatum in esophageal cancer tissue is associated with prognosis. Clin Cancer Res. 2016;22(22):5574–5581. doi:10.1158/1078-0432.CCR-16-1786. 27769987

[167] Xue Y, Xiao H, Guo S, Xu B, Liao Y, Wu Y, Zhang G. Indoleamine 2,3-dioxygenase expression regulates the survival and proliferation of Fusobacterium nucleatum in THP-1-derived macrophages. Cell Death Dis. 2018;9(3):355. doi:10.1038/s41419-018-0389-0. 29500439 PMC5834448

[168] Kook J-K, Park S-N, Lim YK, Cho E, Jo E, Roh H, Shin Y, Paek J, Kim H-S, Kim H, et al. Correction to: genome-based reclassification of Fusobacterium nucleatum subspecies at the species level. Curr Microbiol. 2021;79(1):2. doi:10.1007/s00284-021-02680-w. 34878597

[169] Parte AC, Sardà Carbasse J, Meier-Kolthoff JP, Reimer LC, Göker M. List of prokaryotic names with standing in nomenclature (LPSN) moves to the DSMZ. Int J Syst Evol Microbiol. 2020;70(11):5607–5612. doi:10.1099/ijsem.0.004332. 32701423 PMC7723251

