# Correction

**DOI:** 10.1080/19490976.2024.2418774

**Published:** 2024-10-25

**Article title**: Reexamining the role of Fusobacterium nucleatum subspecies in clinical and experimental studies

**Authors**: Krieger, M., Guo, M., and Merritt, J.

**Journal**: *KGMI: Gut Microbes*

**DOI**: https://doi.org/10.1080/19490976.2024.2415490

The article was originally published with the incorrect figure 2.

**Figure 2.**
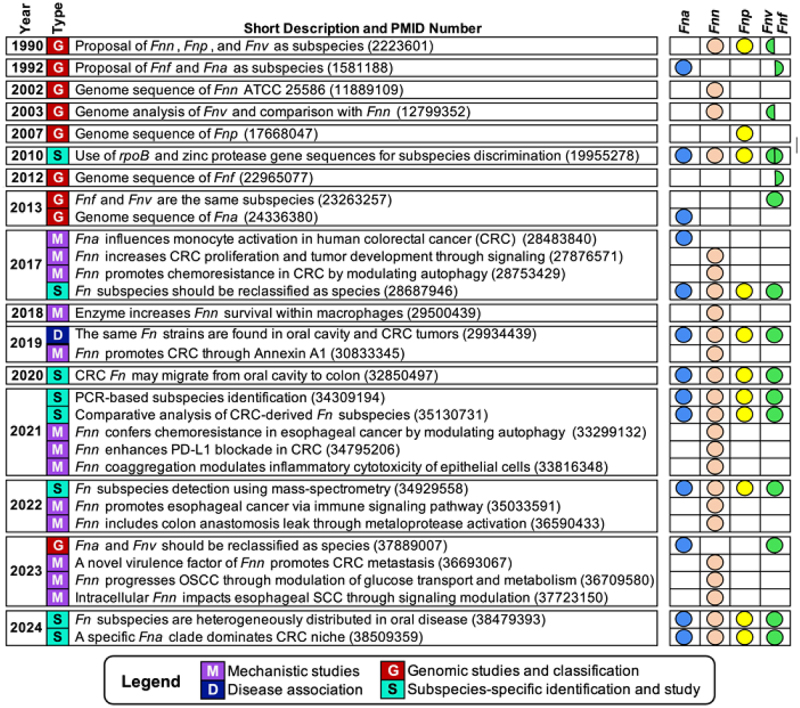
Major milestones in *Fusobacterium nucleatum* subspecies research. Study type is indicated by the color and letter marker in the second column as described in the figure legend. The specific subspecies mentioned in each study is indicated by the colored circles on the right panel (*Fn. animalis* in blue, *Fn. nucleatum* in orange, *Fn. polymorphum* in yellow, and *Fn. fusiforme/vincentii* in green).

The correct version of figure 2 is provided below have been included in the original article, and it has been republished accordingly.

